# Supplementary figures and images for: A novel framework for secure cryptocurrency transactions using quantum crypto guard
Source: PeerJ Comput Sci. 2025 Sep 12;11:e3030. doi: 10.7717/peerj-cs.3030 (PMC12453740; doi:10.7717/peerj-cs.3030)

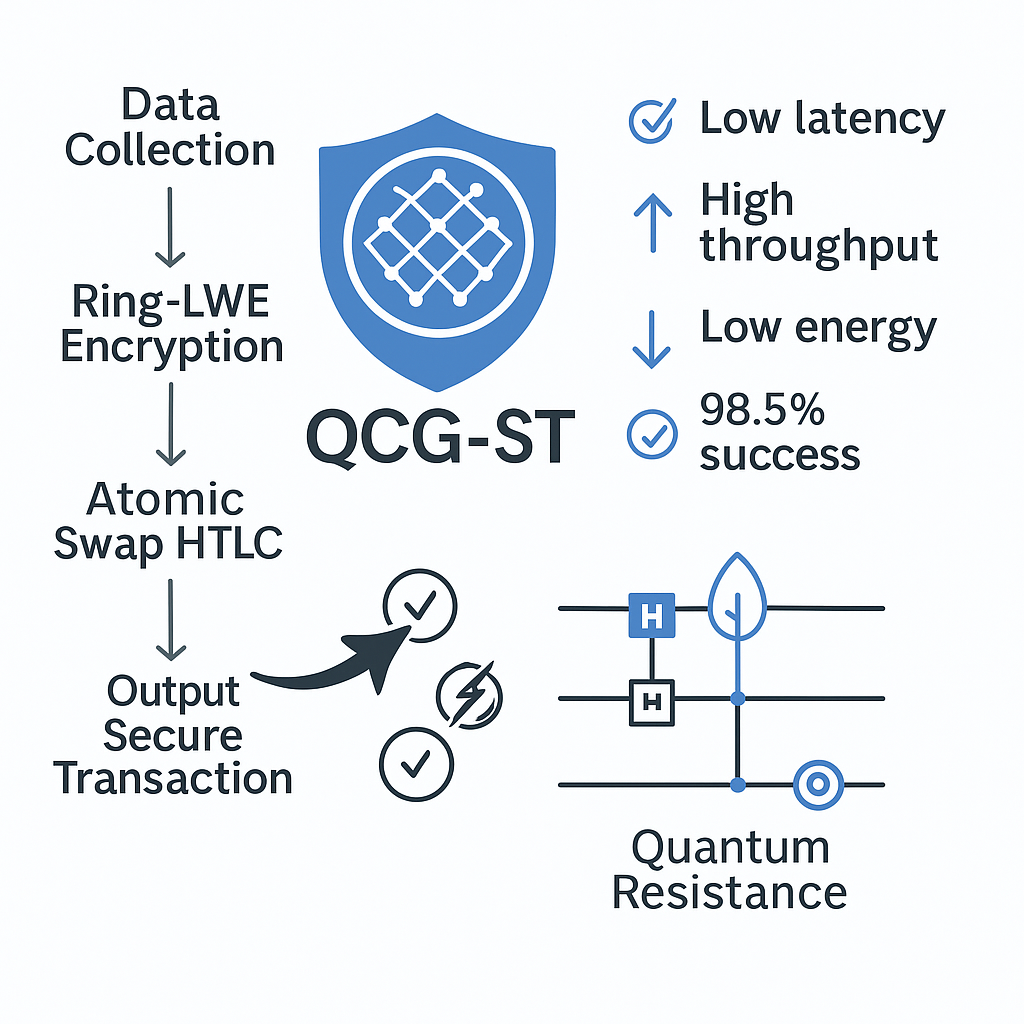

Supplement: Supplemental Information 4 — Blockchain data enter a Ring-LWE-encrypted layer for quantum-resistant security; PoS + sharding + threshold signatures deliver scalable consensus; Zero-Knowledge Proofs assure private validation; a hashed-time-lock atomic-swap path enables cross-chain exchange. Side call-outs highlight QCG-ST’s superior success-rate, latency, throughput, and energy metrics. [file peerj-cs-11-3030-s004.png]
